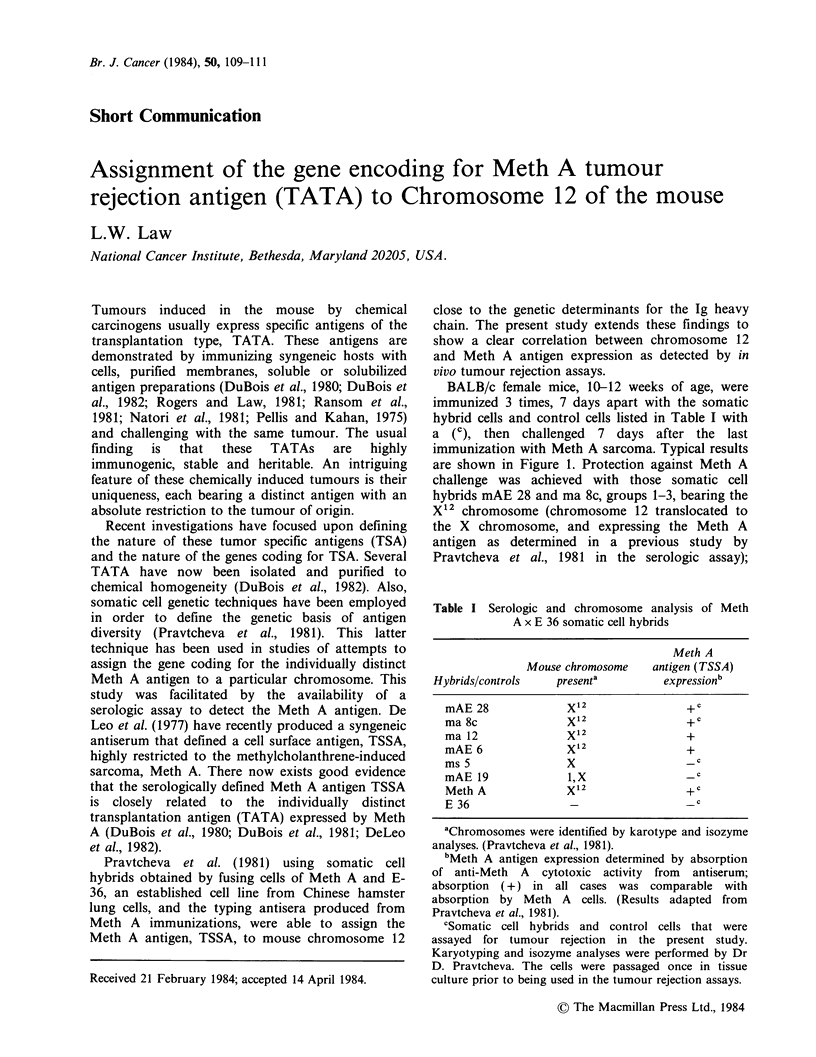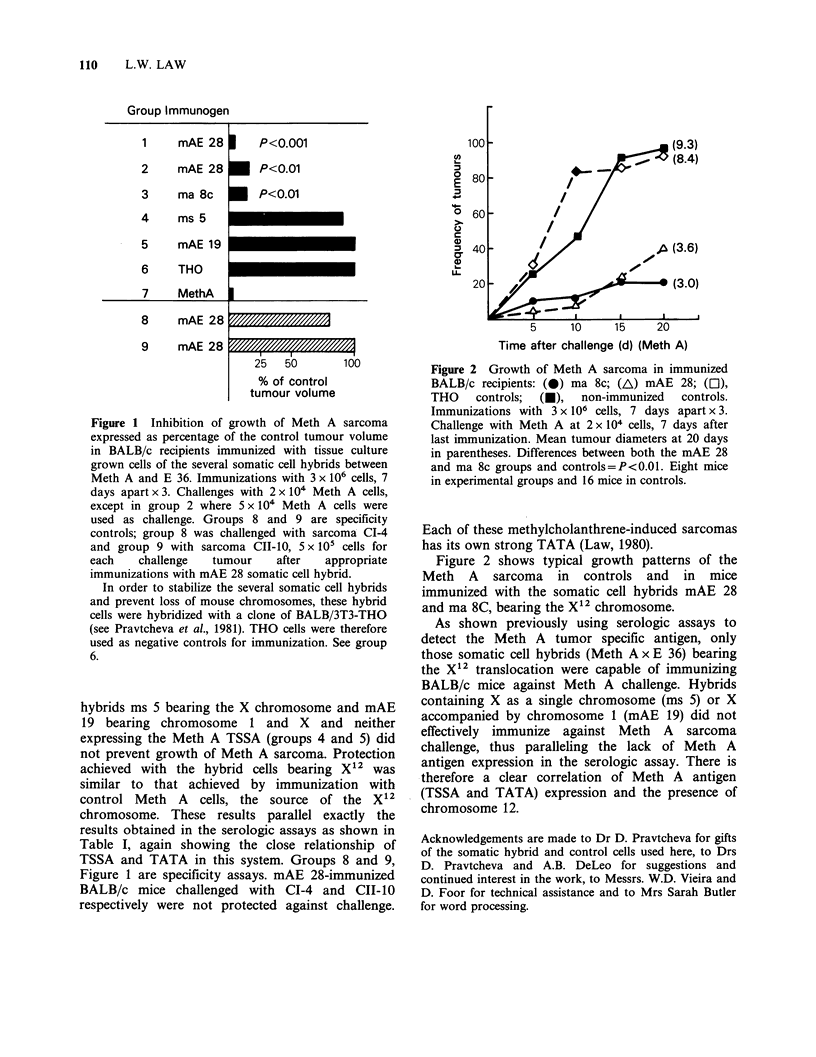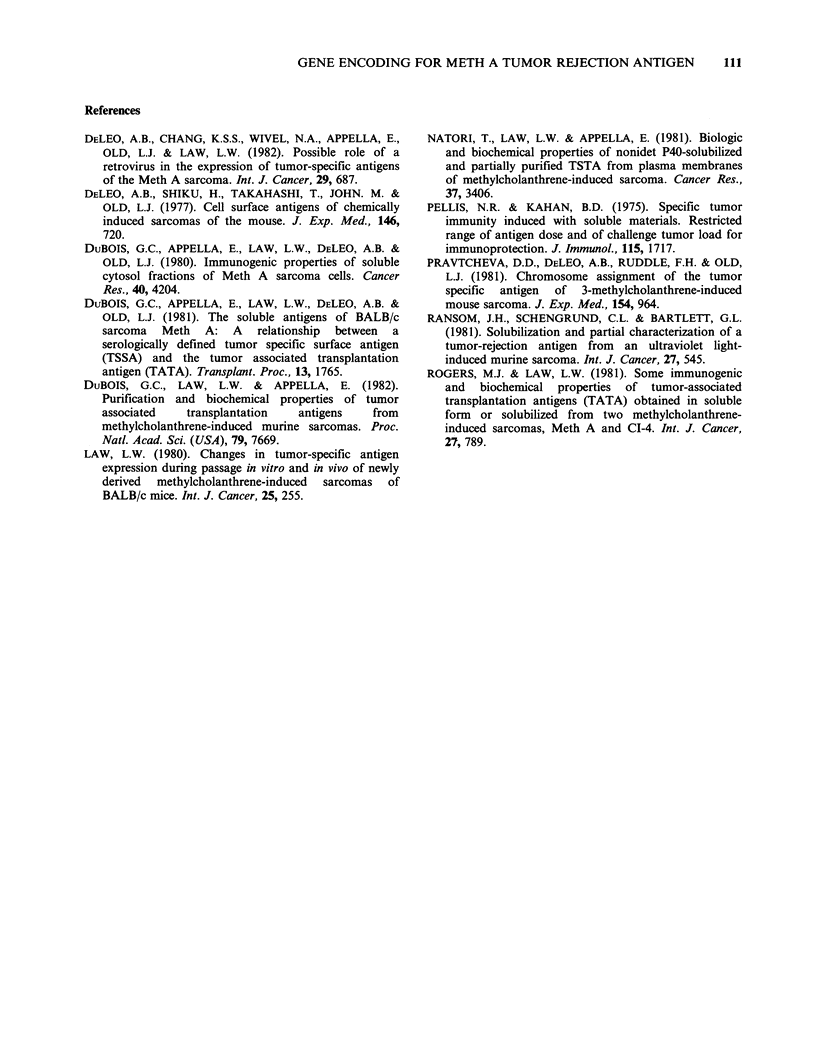# Assignment of the gene encoding for Meth A tumour rejection antigen (TATA) to chromosome 12 of the mouse.

**DOI:** 10.1038/bjc.1984.145

**Published:** 1984-07

**Authors:** L. W. Law


*Br. J. Cancer* (1984), **50**, 109–111

### Short Communication

### Assignment of the gene encoding for Meth A tumour rejection antigen (TATA) to Chromosome 12 of the mouse

L.W. Law

*National Cancer Institute, Bethesda, Maryland 20205, USA.*

Tumours induced in the mouse by chemical carcinogens usually express specific antigens of the transplantation type, TATA. These antigens are demonstrated by immunizing syngeneic hosts with cells, purified membranes, soluble or solubilized antigen preparations (DuBois *et al.*, 1980; DuBois *et al.*, 1982; Rogers and Law, 1981; Ransom *et al.*, 1981; Natori *et al.*, 1981; Pellis and Kahan, 1975) and challenging with the same tumour. The usual finding is that these TATAs are highly immunogenic, stable and heritable. An intriguing feature of these chemically induced tumours is their uniqueness, each bearing a distinct antigen with an absolute restriction to the tumour of origin.

Recent investigations have focused upon defining the nature of these tumor specific antigens (TSA) and the nature of the genes coding for TSA. Several TATA have now been isolated and purified to chemical homogeneity (DuBois *et al.*, 1982). Also, somatic cell genetic techniques have been employed in order to define the genetic basis of antigen diversity (Pravtcheva *et al.*, 1981). This latter technique has been used in studies of attempts to assign the gene coding for the individually distinct Meth A antigen to a particular chromosome. This study was facilitated by the availability of a serologic assay to detect the Meth A antigen. De Leo *et al.* (1977) have recently produced a syngeneic antiserum that defined a cell surface antigen, TSSA, highly restricted to the methylcholanthrene-induced sarcoma, Meth A. There now exists good evidence that the serologically defined Meth A antigen TSSA is closely related to the individually distinct transplantation antigen (TATA) expressed by Meth A (DuBois *et al.*, 1980; DuBois *et al.*, 1981; DeLeo *et al.*, 1982).

Pravtcheva *et al.* (1981) using somatic cell hybrids obtained by fusing cells of Meth A and E-36, an established cell line from Chinese hamster lung cells, and the typing antisera produced from Meth A immunizations, were able to assign the Meth A antigen, TSSA, to mouse chromosome 12 close to the genetic determinants for the Ig heavy chain. The present study extends these findings to show a clear correlation between chromosome 12 and Meth A antigen expression as detected by *in vivo* tumour rejection assays.

BALB/c female mice, 10–12 weeks of age, were immunized 3 times, 7 days apart with the somatic hybrid cells and control cells listed in Table I with a ($^c$), then challenged 7 days after the last immunization with Meth A sarcoma. Typical results are shown in Figure 1. Protection against Meth A challenge was achieved with those somatic cell hybrids mAE 28 and ma 8c, groups 1–3, bearing the $X^{12}$ chromosome (chromosome 12 translocated to the X chromosome, and expressing the Meth A antigen as determined in a previous study by Pravtcheva *et al.*, 1981 in the serologic assay);

**Table I** Serologic and chromosome analysis of Meth A × E 36 somatic cell hybrids

| *Hybrids/controls* | *Mouse chromosome present*[a] | *Meth A antigen (TSSA) expression*[b] |
|---|---|---|
| mAE 28 | $X^{12}$ | +[c] |
| ma 8c | $X^{12}$ | +[c] |
| ma 12 | $X^{12}$ | + |
| mAE 6 | $X^{12}$ | + |
| ms 5 | X | −[c] |
| mAE 19 | 1,X | −[c] |
| Meth A | $X^{12}$ | +[c] |
| E 36 | − | −[c] |

[a]Chromosomes were identified by karotype and isozyme analyses. (Pravtcheva *et al.*, 1981).

[b]Meth A antigen expression determined by absorption of anti-Meth A cytotoxic activity from antiserum; absorption (+) in all cases was comparable with absorption by Meth A cells. (Results adapted from Pravtcheva *et al.*, 1981).

[c]Somatic cell hybrids and control cells that were assayed for tumour rejection in the present study. Karyotyping and isozyme analyses were performed by Dr D. Pravtcheva. The cells were passaged once in tissue culture prior to being used in the tumour rejection assays.

Received 21 February 1984; accepted 14 April 1984.

© The Macmillan Press Ltd., 1984

**Figure 1** Inhibition of growth of Meth A sarcoma expressed as percentage of the control tumour volume in BALB/c recipients immunized with tissue culture grown cells of the several somatic cell hybrids between Meth A and E 36. Immunizations with $3 \times 10^6$ cells, 7 days apart $\times$ 3. Challenges with $2 \times 10^4$ Meth A cells, except in group 2 where $5 \times 10^4$ Meth A cells were used as challenge. Groups 8 and 9 are specificity controls; group 8 was challenged with sarcoma CI-4 and group 9 with sarcoma CII-10, $5 \times 10^5$ cells for each challenge tumour after appropriate immunizations with mAE 28 somatic cell hybrid.

In order to stabilize the several somatic cell hybrids and prevent loss of mouse chromosomes, these hybrid cells were hybridized with a clone of BALB/3T3-THO (see Pravtcheva *et al.*, 1981). THO cells were therefore used as negative controls for immunization. See group 6.

hybrids ms 5 bearing the X chromosome and mAE 19 bearing chromosome 1 and X and neither expressing the Meth A TSSA (groups 4 and 5) did not prevent growth of Meth A sarcoma. Protection achieved with the hybrid cells bearing $X^{12}$ was similar to that achieved by immunization with control Meth A cells, the source of the $X^{12}$ chromosome. These results parallel exactly the results obtained in the serologic assays as shown in Table I, again showing the close relationship of TSSA and TATA in this system. Groups 8 and 9, Figure 1 are specificity assays. mAE 28-immunized BALB/c mice challenged with CI-4 and CII-10 respectively were not protected against challenge.

**Figure 2** Growth of Meth A sarcoma in immunized BALB/c recipients: (●) ma 8c; (△) mAE 28; (□), THO controls; (■), non-immunized controls. Immunizations with $3 \times 10^6$ cells, 7 days apart $\times$ 3. Challenge with Meth A at $2 \times 10^4$ cells, 7 days after last immunization. Mean tumour diameters at 20 days in parentheses. Differences between both the mAE 28 and ma 8c groups and controls = $P < 0.01$. Eight mice in experimental groups and 16 mice in controls.

Each of these methylcholanthrene-induced sarcomas has its own strong TATA (Law, 1980).

Figure 2 shows typical growth patterns of the Meth A sarcoma in controls and in mice immunized with the somatic cell hybrids mAE 28 and ma 8C, bearing the $X^{12}$ chromosome.

As shown previously using serologic assays to detect the Meth A tumor specific antigen, only those somatic cell hybrids (Meth A $\times$ E 36) bearing the $X^{12}$ translocation were capable of immunizing BALB/c mice against Meth A challenge. Hybrids containing X as a single chromosome (ms 5) or X accompanied by chromosome 1 (mAE 19) did not effectively immunize against Meth A sarcoma challenge, thus paralleling the lack of Meth A antigen expression in the serologic assay. There is therefore a clear correlation of Meth A antigen (TSSA and TATA) expression and the presence of chromosome 12.

Acknowledgements are made to Dr D. Pravtcheva for gifts of the somatic hybrid and control cells used here, to Drs D. Pravtcheva and A.B. DeLeo for suggestions and continued interest in the work, to Messrs. W.D. Vieira and D. Foor for technical assistance and to Mrs Sarah Butler for word processing.

### References

DeLeo, A.B., Chang, K.S.S., Wivel, N.A., Appella, E., Old, L.J. & Law, L.W. (1982). Possible role of a retrovirus in the expression of tumor-specific antigens of the Meth A sarcoma. *Int. J. Cancer*, **29**, 687.

DeLeo, A.B., Shiku, H., Takahashi, T., John. M. & Old, L.J. (1977). Cell surface antigens of chemically induced sarcomas of the mouse. *J. Exp. Med.*, **146**, 720.

DuBois, G.C., Appella, E., Law, L.W., DeLeo, A.B. & Old, L.J. (1980). Immunogenic properties of soluble cytosol fractions of Meth A sarcoma cells. *Cancer Res.*, **40**, 4204.

DuBois, G.C., Appella, E., Law, L.W., DeLeo, A.B. & Old, L.J. (1981). The soluble antigens of BALB/c sarcoma Meth A: A relationship between a serologically defined tumor specific surface antigen (TSSA) and the tumor associated transplantation antigen (TATA). *Transplant. Proc.*, **13**, 1765.

DuBois, G.C., Law, L.W. & Appella, E. (1982). Purification and biochemical properties of tumor associated transplantation antigens from methylcholanthrene-induced murine sarcomas. *Proc. Natl. Acad. Sci. (USA)*, **79**, 7669.

Law, L.W. (1980). Changes in tumor-specific antigen expression during passage *in vitro* and *in vivo* of newly derived methylcholanthrene-induced sarcomas of BALB/c mice. *Int. J. Cancer*, **25**, 255.

Natori, T., Law, L.W. & Appella, E. (1981). Biologic and biochemical properties of nonidet P40-solubilized and partially purified TSTA from plasma membranes of methylcholanthrene-induced sarcoma. *Cancer Res.*, **37**, 3406.

Pellis, N.R. & Kahan, B.D. (1975). Specific tumor immunity induced with soluble materials. Restricted range of antigen dose and of challenge tumor load for immunoprotection. *J. Immunol.*, **115**, 1717.

Pravtcheva, D.D., DeLeo, A.B., Ruddle, F.H. & Old, L.J. (1981). Chromosome assignment of the tumor specific antigen of 3-methylcholanthrene-induced mouse sarcoma. *J. Exp. Med.*, **154**, 964.

Ransom, J.H., Schengrund, C.L. & Bartlett, G.L. (1981). Solubilization and partial characterization of a tumor-rejection antigen from an ultraviolet light-induced murine sarcoma. *Int. J. Cancer*, **27**, 545.

Rogers, M.J. & Law, L.W. (1981). Some immunogenic and biochemical properties of tumor-associated transplantation antigens (TATA) obtained in soluble form or solubilized from two methylcholanthrene-induced sarcomas, Meth A and CI-4. *Int. J. Cancer*, **27**, 789.